# Recovery and stress of control center dispatchers in the first waves of the SARS-CoV-2 pandemic

**DOI:** 10.1007/s00508-022-02144-6

**Published:** 2023-01-04

**Authors:** Heiko Schumann, Beatrice Thielmann, Julia Botscharow, Irina Böckelmann

**Affiliations:** Institute of Occupational Medicine, School of Medicine, Otto von Guericke University Magdeburg, Leipziger Str. 44, 39120 Magdeburg, Germany

**Keywords:** Mental stress, Controller, Emergency medical services, COVID-19 pandemic, Well-being

## Abstract

Control center dispatchers (CCDs) are exposed to high levels of work-related mental stress, which are exacerbated by the current severe acute respiratory syndrome coronavirus 2 (SARS-CoV-2) pandemic. The aim of this study was to comparatively analyze the recovery and stress state of CCDs during the first and second waves of the SARS-CoV‑2 pandemic. A total of 490 CCDs (*n* = 440, t1 and *n* = 50, t2) with a mean age of 42.26 ± 8.79 years participated directly at the end of the first wave from June to August 2020 (t1) and during the second wave between January and February 2021 (t2) of the SARS-CoV‑2 pandemic. The short form (EBF-24/A; test form S2) of the Kallus recovery-stress questionnaire (EBF) was applied. Over the course of the two survey phases, the dimensions strain significantly increased (t1: 2.47 ± 1.08 vs. t2: 3.12 ± 0.93 points, *p* < 0.001) and recovery significantly decreased (t1: 3.03 ± 0.94 vs. t2: 2.50 ± 0.81 points, *p* < 0.001). Significant to highly significant differences were present in the EBF dimensions of the two waves in the majority of cases, even when taking the waves into account. For the variable “recovery in the social field”, a medium effect was noticeable in the corrected model (η^2^ = 0.064). The SARS-CoV‑2 pandemic illustrates that the stress experience increased from the first to the second waves, and the recovery of CCDs decreased. These data provide a directional trend as the pandemic is ongoing, and stress and strain situations in control centers may continue to worsen. Immediate health promotion and prevention measures are essential.

## Introduction

Work in an emergency control center is often unpredictable and can be cognitively, emotionally, and physically demanding [1]. Control center dispatchers (CCDs) have been exposed to particularly high levels of additional mental stress since the severe acute respiratory syndrome coronavirus 2 (SARS-CoV-2) pandemic began in the spring of 2020 [1].

Before the ambulance team of an emergency service can drive to the emergency scene, an emergency call is first reported to an emergency control center for processing. The CCDs perform a telephone triage of the emergency call, which presents several unique challenges. They must aurally perceive and subjectively evaluate the situation quickly without visually assessing what is happening and thus must rely on a caller’s descriptions. In doing so, they must gather as much information as possible, in a targeted manner to assess the urgency of the call. Relevant information in the sense of a mission cue is then forwarded to the rescue service’s response team. If immediate life-saving measures are necessary, telephone resuscitation is initiated, whereby callers and first responders are instructed to perform life-saving measures [2]. This certainly carries professional and social responsibility [3].

A review identified occupational stressors of CCDs, although the authors also pointed out the need for further research studies among CCDs [3]. The stressors included traumatic and emotional events, not having control over high workloads, a lack of management support, and working under time pressure [3]. In contrast, support from friends and family as a psychosocial resource was helpful for CCDs in coping with these work-related stressors [3]. Another study showed that rapid and time-limited risk assessments, decision making, and coping with unexpected developments were common sources of stress [4].

Annual full surveys of the operation numbers of the federal states in Germany show a growth rate of approximately 5% per year in rescue service operations, and it can be concluded that the numbers of emergency calls to the control centers are also increasing [5]. An increase in the use of rescue services can also be observed worldwide [6, 7]. In addition, the current SARS-CoV‑2 pandemic has led to an increase in ambulance calls [8], although these could also decrease in some regions in times of lockdown [9]. The workloads of CCDs in Germany increased during the pandemic for 88% of the surveyed CCDs, and job satisfaction decreased for 56% [1].

Working under stressful conditions and repeated confrontation with difficult situations can have a negative impact on the physical and psychological health of CCDs, especially if stress compensation is inadequate [10]. The basic theoretical model of the development of health impairments as a result of occupational stressful situations in this study represents the stress–strain concept [11–13]. The construct of (work) stress and the available organizational, psychosocial, and personal resources for coping with the stress result in a psychological state of stress. This state of stress can persist for some time after the stressful situation has ended and lead to exhaustion due to an increasing number of loads [11, 12]. Stress is thus an interaction between an individual and their environment according to the subjective perception and evaluation of stressors [14]. Personality traits also play a special role [14].

Overall, work-related stress remains a major problem for occupational health, yet healthcare workers are now particularly vulnerable under pandemic conditions [15].

It should be noted that little literature is available on the recovery and stress of CCDs during the first waves of the SARS-CoV‑2 pandemic. Thus, the aim of this study was to comparatively analyze the recovery-stress status of CCDs during the first and second waves of the SARS-CoV‑2 pandemic.

## Material and methods

During the SARS-CoV‑2 pandemic, a study on the stress and strain of dispatchers in control centers was conducted, and the recovery-stress conditions were surveyed. In Germany, a minimum qualification of emergency medical technician (German “Rettungssanitäter”) experience is required to complete the advanced qualification to be a control center dispatcher. In this study, more than 80–90% of the respondents were paramedics or emergency paramedics who had a higher professional qualification than emergency medical technician experience.

For this purpose, a voluntary and anonymous cross-sectional online survey at two different time points was conducted throughout Germany between 2020 and 2021 in the first two waves of the pandemic using standardized survey instruments. The time periods for the surveys were from June to August 2020 (t1) and the second wave between January and February 2021 (t2) of the SARS-CoV‑2 pandemic. The survey was conducted directly at the end of the first and during the second wave. A wave is defined as a wave-like occurrence of viral illness in the context of a pandemic (after a decrease, a renewed increase of infection numbers). The presence of certain virus variants plays a role in each wave. Other reasons for the waves include changing symptoms, mortality, or the presence of vaccines [16]. In Germany, these were defined for the periods March–April 2020 (first wave) and October 2020–January 2021 (second wave) [17]. The CCDs were recruited via various social media platforms and the professional German journal “*Rettungsdienst*” (S + K Publisher), which made it impossible to determine the response rate. A positive vote from the ethics committee of the medical faculty of Otto von Guericke University Magdeburg was received.

### Typical characteristics of the first two waves

The first wave was considered the greatest disaster after the World Wars, severely affecting all aspects of life. It was a major public health challenge and social and economic activities around the world were disrupted. The production of goods came to a halt almost everywhere, and the unemployment rate and short-time workers increased. Other social problems occurred, such as family violence and lack of social contact between children due to prolonged stay at home. Consequences of the virus pandemic were almost unknown, and no vaccine was available [16, 18].

During the second wave, there were changes in priorities that were set politically. These decisions varied from region to region, and state to state. In Germany, for example, the instructions were very sharp at the beginning of the pandemic. During the second wave, the priority was set more on economic activity. There was an attempt to balance successful medical care and a growing economy. Despite the balancing act, there was a drastic increase in infections, which in turn led to severe restrictions on outdoor activities, mandatory face masks, and the prevention of human gatherings resulted [16, 18].

### Subjects

The total number of participants was 490 CCDs aged 22–63 years. A total of 440 CCDs (406 males, 34 females) participated during the first wave and 50 (47 males, 3 females) participated during the second wave. Reasons for the low participation in the survey of the second wave are to be assumed in the already existing high and increasing stresses in the professional and private context of the emergency service personnel. The exclusion criteria during the study survey were a lack of paramedic or emergency medical technician training, a lack of training courses to become a dispatcher, and a lack of full-time employment in the dispatch center.

### Methodology

**Fig. 1:**
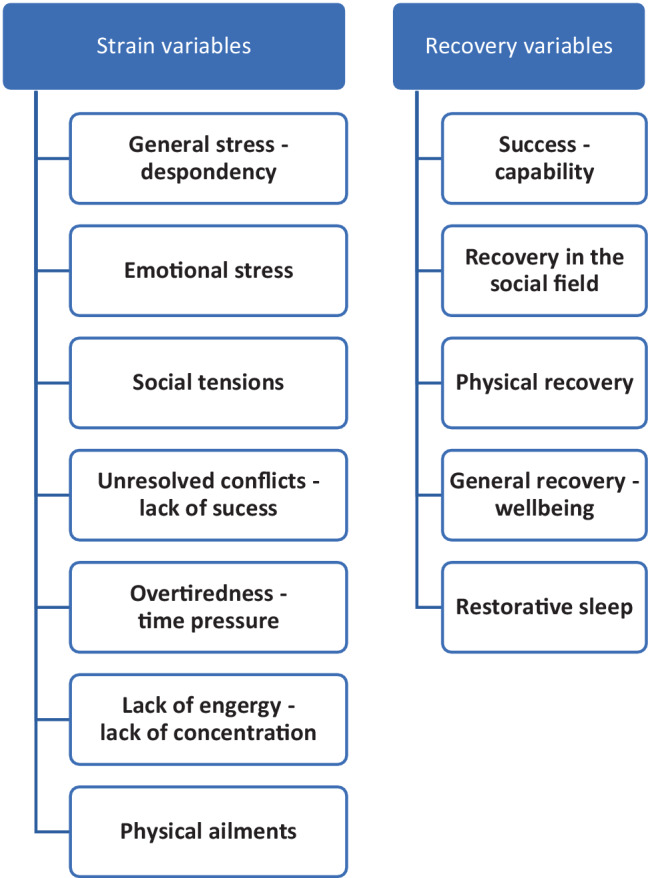
Subscales of the recovery-stress questionnaire according to Kallus [11]

For the research question, the recovery stress questionnaire (EBF, in German *Erholungs-Belastungs-Fragebogen*) according to Kallus [11] was used in its short form (S2). The questionnaire is divided into the “stress” and “recovery” dimensions and the seven and five subscales, respectively (Fig. 1). This made it possible to determine an individual’s current recovery-stress state based on 25 items regarding stressful situations and reactions to them and recovery phases of the last 3 days and nights. The response was based on a 7-point scale ranging from 0 (never) to 6 (all the time).

Mean scores were calculated for the dimensions and their associated subscales. High values indicated high stress or high recovery.

### Statistical analysis

In the first step, the online data were transferred to the psychodiagnostic Vienna system (Schuhfried, Mödling, Austria) and then analyzed by computer. The software SPSS 26® for Windows (IBM, Armonk, NY, USA) was used for the statistical analysis. T tests for normally distributed interval-scaled variables and the Mann–Whitney test for nonnormally distributed interval-scaled variables and ordinal variables were used, as due to the anonymity of the online survey it could not be concluded that all respondents in the second wave also participated in the first pandemic wave. Further analyses of the data were carried out using the general linear model (ALM) and χ^2^-tests. A multifactorial analysis of variance/ANOVA was used to analyze the influence of the variables sex, age and pandemic wave on the “recovery” and “stress” dimensions.

## Results

### Sociodemographic and occupational data

A total of 490 CCDs participated in the study, with a mean age of 42.26 ± 8.79 years. During the first wave, the mean age was 42.43 ± 8.75 years, and at the time of the second wave, it was 40.82 ± 9.21 years, which was not significantly different (*p* = 0.605). The sex distribution of the participants, such as the ages of the participants of both sexes, was statistically comparable during both waves.

### Recovery-stress state

To assess the scale reliability, the internal consistency was calculated. The Cronbach’s alpha of the dimension “stress” (α = 0.917) could be assessed as excellent and that of the dimension “recovery” (α = 0.851) as good. Thus, the reliability was confirmed.

Table 1 demonstrates the differences between the first two waves. Except for the “unresolved conflicts—lack of success” and “success—performance” subscales, there were significant to highly significant differences in all scales, including the superordinate dimensions. When looking at the mean values, it was also clear that all subscales of the dimension “stress” had higher values in the second wave. In contrast, all scales of the dimension “recovery” were lower in this wave.

**Table 1:** EBF characteristics of the samples in both surveys

EBF variables	1st wave	2nd wave	p_Mann-Whitney test_
–	*MW* *±* *SD*		–
	*Median (Min–Max)*		
	*[95% CI]*		
Strain	2.47 ± 1.08	3.12 ± 0.93	**<0.001**
	2.36 (0.36–5.86)	3.14 (1.14–4.93)	
	[2.37–2.57]	[2.86–3.39]	
General stress—despondency	2.33 ± 1.48	3.19 ± 1.23	**<0.001**
	2.00 (0–6)	3.50 (0.5–5.5)	
	[2.19–2.47]	[2.84–3.54]	
Emotional stress	2.58 ± 1.24	3.41 ± 1.04	**<0.001**
	2.50 (0.5–6)	3.50 (1–5.5)	
	[2.47–2.70]	[3.11–3.71]	
Social tensions	2.72 ± 1.32	3.48 ± 1.16	**<0.001**
	2.50 (0–6)	3.50 (1–5.5)	
	[2.59–2.84]	[3.15–3.81]	
Unresolved conflicts—lack of success	2.63 ± 1.29	2.95 ± 1.32	0.109
	2.50 (0–6)	3.00 (0.5–5)	
	[2.51–2.75]	[2.57–3.33]	
Overtiredness—time pressure	2.78 ± 1.42	3.55 ± 1.18	**<0.001**
	2.50 (0–6)	4.00 (1–5)	
	[2.64–2.91]	[3.22–3.89]	
Lack of energy—lack of concentration	2.08 ± 1.25	2.61 ± 1.31	**0.005**
	2.00 (0–6)	2.50 (0.5–5.5)	
	[1.97–2.20]	[2.24–2.98]	
Physical ailments	2.15 ± 1.26	2.68 ± 1.24	**0.003**
	2.00 (0–6)	2.50 (0.5–5)	
	[2.03–2.27]	[2.33–3.03]	
Recovery	3.03 ± 0.94	2.50 ± 0.81	**<0.001**
	3.00 (0.8–5.6)	2.30 (0.8–4.7)	
	[2.94–3.12]	[2.27–2.74]	
Success—capability	3.32 ± 1.09	3.15 ± 1.04	0.271
	3.50 (0.5–6)	3.25 (0.5–5.5)	
	[3.22–3.42]	[2.86–3.45]	
Recovery in the social field	2.77 ± 1.10	2.10 ± 0.90	**<0.001**
	2.50 (0–5.5)	2.00 (0.5–5)	
	[2.66–2.87]	[1.84–2.36]	
Physical recovery	2.88 ± 1.16	2.29 ± 1.00	**<0.001**
	3.00 (0.5–6)	2.00 (0.5–5)	
	[2.77–2.98]	[2.01–2.57]	
General recovery—well-being	3.28 ± 1.15	2.64 ± 1.04	**<0.001**
	3.50 (0.5–6)	2.50 (1–5)	
	[3.18–3.39]	[2.34–2.94]	
Restorative sleep	2.90 ± 1.44	2.34 ± 1.23	**0.008**
	3.00 (0–6)	2.50 (0.5–5)	
	[2.76–3.03]	[1.99–2.69]	

The significant *p*-values are marked in bold

EBF point scale ranges from 0 (never) to 6 (all the time)

*EBF* recovery stress questionnaire (in German Erholungs-Belastungs-Fragebogen)

The results of the multifactorial analyses of variance are shown in Table 2. Highly significant differences with *p* < 0.001 were found for the EBF variables “strain” (η^2^ = 0.045), “general stress—despondency” (η^2^ = 0.041), “emotional stress” (η^2^ = 0.052), “social tensions” (η^2^ = 0.064), and “recovery in the social field” (η^2^ = 0.064).

**Table 2:** EBF subscales considering gender, age, and pandemic wave from analysis of variance with assessment of effect size (η^2^)

	Corrected model			Gender		Age		Wave	
*EBF variables*	*F*	*Sig*	*η*^*2*^	*p*	*η*^*2*^	*p*	*η*^*2*^	*p*	*η*^*2*^
Strain	7.680	**<** **0.001**	0.045	**0.026**	0.010	0.348	0.002	**<** **0.001**	0.034
General stress—despondency	6.900	**<** **0.001**	0.041	**0.033**	0.009	0.557	0.001	**<** **0.001**	0.031
Emotional stress	8.937	**<** **0.001**	0.052	0.071	0.007	0.107	0.005	**<** **0.001**	0.040
Social tensions	8.347	**<** **0.001**	0.049	0.083	0.006	**0.013**	0.013	**<** **0.001**	0.030
Unresolved conflicts—lack of success	1.683	0.170	0.010	0.200	0.003	0.399	0.001	0.087	0.006
Overtiredness—time pressure	5.558	**0.001**	0.033	0.103	0.005	0.591	0.001	**<** **0.001**	0.027
Lack of energy—lack of concentration	3.178	**0.024**	0.019	0.204	0.003	0.885	< 0.001	**0.005**	0.016
Physical ailments	5.552	**0.001**	0.033	**0.006**	0.016	0.377	0.002	**0.005**	0.016
Recovery	5.884	**0.001**	0.035	0.324	0.002	0.126	0.005	**<** **0.001**	0.030
Success—capability	1.113	0.343	0.007	0.523	0.001	0.182	0.004	0.263	0.003
Recovery in the social field	11.039	**<** **0.001**	0.064	0.124	0.005	**<** **0.001**	0.026	**<** **0.001**	0.039
Physical recovery	4.036	**0.007**	0.024	0.538	0.001	0.896	< 0.001	**0.001**	0.024
General recovery—well-being	5.176	**0.002**	0.031	0.868	< 0.001	0.289	0.002	**<** **0.001**	0.030
Restorative sleep	3.579	**0.014**	0.022	0.054	0.008	0.756	< 0.001	**0.008**	0.015

Partial η^2^ < 0.06 corresponds to a small effect, partial η^2^ 0.06–0.14 corresponds to a medium effect, and partial η^2^ > 0.14 corresponds to a large effect

The significant *p*-values are marked in bold.

*EBF* recovery stress questionnaire (in German Erholungs-Belastungs-Fragebogen)

Significant differences between men and women were only found for the “strain” (*p* = 0.026, η^2^ = 0.010), “general stress—despondency” (*p* = 0.033, η^2^ = 0.009) and “physical ailments” scales (*p* = 0.006, η^2^ = 0.016). Regarding age, significant to highly significant differences only occurred in the “social tension” (*p* = 0.013, η^2^ = 0.013), and “recovery in the social field” (*p* < 0.01, η^2^ = 0.026) categories. A medium effect became visible in the corrected model only for the variable “recovery in the social field” (η^2^ = 0.064). In all other EBF variables, there were only small effects.

## Discussion

This study focused on subjective perceptions of recovery and stress among full-time control center dispatchers during the first two waves of SARS-CoV‑2 pandemic. The pandemic is associated with an increase in stress and strain on healthcare workers, including emergency service workers [8, 19]. Data on the recovery and strain of CCDs are limited to date. Preliminary figures show increased workloads and decreased job satisfaction over the course of the initial pandemic waves [1]. In this study, it was shown that subjectively perceived stress increased, but recovery decreased. Examples for increased variables are “general stress—despondency”, “emotional stress”, “social tensions”, and “overtiredness—time pressure”. Significantly decreased variables were “recovery in the social field”, “physical recovery”, and “general recovery—well-being”. With the help of a multifactorial analysis of variance, only a minor influence of the sex, age and wave variables on the “recovery” and “strain” dimensions of the EBF could be analyzed. The power of the results is minor because the effect size is mostly low.

A review showed that the psychological effects following lockdowns are small and highly heterogeneous [20]. Our collected data also confirm these findings due to the low effect sizes of the wave variables on the stress and recovery dimensions. A study from Tyrol, Austria, investigated a common practice during the main times of the pandemic in the spring of 2020 by initially only alerting a reduced emergency rescue team to emergency scenes to maintain the structure of care of the rescue service by reducing contact with people at the scene of the emergency [21]. This usually involved first delegating ambulance vehicles to the scene. The emergency physician reassignment rate of the reduced missions was 14.5%. However, the authors concluded that if the saved emergency physician calls had been taken into account, there would have been an overall increase in emergency physician calls compared with previous years [21]. In this context, the frequency of missions with respiratory distress increased to twice as often in 2020 compared to 2017–2019 [21]. It can be surmised that these reduced call-outs could also mean that there was additional work for CCDs, as emergency physicians who might have been prealerted were 14.5% reordered. Here, a role conflict may also emerge as a strain, as the best possible care for a patient could not be guaranteed [19]. This role conflict can lead to negative thoughts about oneself or others, as well as intense feelings of guilt or shame [22]. This could be indicated by the EBF “emotional stress” dimension in our data.

The present study showed no sex differences and only small effects of sex on the dimensions of strain and recovery. This may be due to the low proportion of women (7.6%) in our study. One review concluded that fear of the unknown or of contracting the SARS-CoV‑2 virus were strong psychological stressors, and it was a greater risk for female caregivers [23]. Another meta-analysis also indicated that female healthcare workers had higher rates of affective symptoms than male employees [24].

Age also had little influence on the results. Other studies have indicated that subjective perceptions of psychosocial workload increase with age, especially among older men [25]. Another study demonstrated an age-related effect on occupational ambition [26]. Here, role conflict may become problematic because professional ambition is presumably incompatible with a reduced assignment measure.

A limiting factor in assessing the results is that regional differences depending on the extent of the pandemic were not considered. The passive recruitment of subjects used in this online survey can lead to a selection bias, as only those people who are interested in the topic of the survey participate—especially when one is highly stressed. The time period chosen for the survey could also lead to bias, as circumstances at the beginning of the wave in question may not be remembered. Thus, in this online survey, it could not be determined with certainty that any CCDs from particularly stressed regions participated in the study, which could also indicate lower participation during the second wave. Similarly, no circumstances from the private sphere were taken into account, e.g., personal COVID-19 infection, home schooling children, a lack of childcare or care for relatives, which may well lead to an increased sense of stress. Other additional workloads that contributed to the results include increased hygiene requirements and working with patients under special personal protective equipment [29]. Also longer working hours, which can reduce private life and thus social support, are rated as a missing resource [29]. The loss of colleagues to quarantine and illness exacerbates the shortage of qualified emergency medical services personnel that has already existed for years. During the first two waves of the pandemic, vaccines were not available or vaccination against the SARS-CoV‑2 virus was not mandatory. Here, mismatches are possible between vaccinated and non-vaccinated personnel. The proportion of “vaccine refusers” in the workplace could be relevant because there could be fear of colleagues not being vaccinated and thus transmitting disease. Other external factors can also play a role. News from media (e.g. newspapers, radio, TV) transported fear contents or news about penalties for broken COVID rules, changing and unsure behavioral rules, quarantine with temporary “loss” of colleagues, frustration due to broken easing promises. The COVID rules in Germany were binding and extensive. In the context of the use of face masks, skin irritations are increasingly reported [31]. In addition to sensitive skin, progression and worsening of the skin are also reported in atopic dermatitis, acne and seborrheic dermatitis. These skin diseases lead to negative effects on sleep, mental health, and quality of life [32]. It is not surprising that the loads listed above, considering lockdowns, led to an increase in stress. Whether a habituation to the mask wearing and increased hygiene effort will occur, must be further investigated.

In summary, the increase in subjective strain with a concomitant decrease in recovery within two SARS-CoV‑2 pandemic waves indicates a directional trend in the current strain situation of CCDs. Further surveys are certainly needed to evaluate the causes of the decrease in recovery and increase in strain.

From the point of view of occupational medicine and health promotion, it is important to create framework conditions that support long-term participation and promote the personal development of competencies as a resource for employees to reduce stress and strain. The aim is to prevent the risk of mental disorders such as anxiety disorders, depression or burnout [27, 28].

Organizational, social, personal, and psychological factors and resources can be considered appropriate measures to support the mental health of emergency service personnel and thus employees in control centers [30]. In this regard, however, there is still a great need for research to determine the effectiveness of these measures [30]. Healthcare workers have both problem-centered and emotion-centered coping strategies to better manage the stressors and situation of the SARS-CoV‑2 pandemic. In this context, coping behaviors, resilience, and social support were associated with positive mental and psychological health outcomes [33].
